# Efficacy and safety of adalimumab biosimilar (HS016) in inflammatory bowel disease from the real-world study

**DOI:** 10.3389/fphar.2023.1259183

**Published:** 2023-10-16

**Authors:** Fang Wang, Xiaofei Li, Yanting Shi, He Zhou, Gang Yang, Ruixia Li, Tong Wu, Jie Liang

**Affiliations:** State Key Laboratory of Holistic Integrative Management of Gastrointestinal Cancers and National Clinical Research Center for Digestive Diseases, Xijing Hospital of Digestive Diseases, Fourth Military Medical University, Xi’an, China

**Keywords:** adalimumab biosimilar, therapeutic effect evaluation, Crohn’s disease, ulcerative colitis, therapeutic drug monitoring

## Abstract

**Objective:** Adalimumab (ADA) is an effective treatment for inflammatory bowel disease (IBD), both ulcerative colitis (UC) and Crohn’s disease (CD). The equal effect between the original ADA and biosimilars from Europe and the United States has been shown. However, the biosimilar of ADA is different in China. The effectiveness and safety data of ADA biosimilar (HS016) in China have yet to be discovered.

**Patients and methods:** 91 patients (75 CD, 16 UC) received HS016 treatment and were enrolled in this study. Therapeutic response and safety profiles were analyzed. Therapeutic drug monitoring (TDM) was also carried out among nonresponse patients. After being considered as “nonresponse” (after three or 6 months of treatment), 20 patients’ serum TNFα concentrations were measured and correlated to their disease severity.

**Results:** Among active CD patients (*n* = 61), 75.4% (46/61) at 12 w, 73.8% (45/61) at 26 w, 50.8% (31/61) at 52 w achieved the clinical response, respectively; 55.7% (34/61) at 12 w, 65.6% (40/61) at 26 w, and 45.9% (28/61) at 52 w achieved clinical remission. The maintained remission rates of CD (*n* = 14) in clinical remission were 100% (14/14) at 12 w, 78.6% (11/14) at 26 w, and 63.6% (7/11) at 52 w, respectively. Among active UC patients, 37.5% (6/16) at 12 w and 50% (8/16) at 26 w achieved clinical response. Total adverse event rates were 5.5% (5/91) during 52-week visits. Due to the inadequate serum drug concentration, 30.4% (7/23) of patients had poor clinical responses. Elevations of serum anti-drug antibodies occurred in one additional patient (4.3%).

**Conclusion:** ADA biosimilar HS016 had good efficacy and safety in Chinese IBD patients.

## 1 Introduction

Inflammatory bowel disease (IBD), including ulcerative colitis (UC) and Crohn’s disease (CD), is a chronic inflammatory disorder of the intestine. The incidence of IBD continually increases worldwide, inducing a tremendous economic burden on patients and society (Kaplan, 2015). Although the disease’s pathogenesis is unclear, it may correlate to genetic susceptibility, environmental factors, and gut dysbiosis. All the disorders above lead to immune imbalance and consequently to the development of diseases, which are usually mediated by Th1 and Th17 cells. They produce large amounts of cytokines such as TNF-α, IL-17, and IL-1β (Romagnani, 1999; Torres et al., 2017), which cause chronic inflammatory response increasing. As the research progresses, traditional treatments (including 5-aminosalicylic acid, glucocorticoids, immunosuppressants, etc.,) have shifted to biological therapy (Pouillon et al., 2020). There has been an increasing number of biologic medications used for IBD treatment, such as monoclonal antibodies or inhibitors to tumor necrosis factor (TNF)-α, interleukin (IL)-12/23, adhesion molecules, and Janus kinase (JAK). Biologics open up a new era of IBD treatment.

During the last 40 years, the cellular and molecular mechanisms of the inflammatory diseases’ pathogenesis have been continuously revealed, including UC, CD, ankylosing spondylitis (AS), rheumatoid arthritis (RA), and psoriasis. Among all inflammatory cytokines and chemokines, TNF-α is the first identified vital factor in developing the inflammatory responding process. Thus anti-TNFα therapy emerged and began to use in RA treatment in the mid-1990 s, and therapeutic goals started to evolve and move forward. Due to its potential effect on disease-modifying and mucosal healing, anti-TNFα therapy is associated with a reduced risk of hospitalization, colectomy, and colorectal cancer, especially among IBD patients. Anti-TNFα acts mainly by binding TNF-α in serum (sTNFα) or on immune cell membranes (mTNFα) (Tracey et al., 2008), and especially the latter is considered as a critical role in biological agents function. Anti-TNFα is the most classic of all biologics, including infliximab (IFX) and adalimumab (ADA). Because of the safety and convenient usage, human-originated and subcutaneous injecting ADA and its biosimilars have been widely used (Sandborn et al., 2007). European Crohn’s and Colitis Organization (ECCO) and the American College of Gastroenterology (ACG) have made it clear that ADA can be used as an option for patients with moderate-severe IBD who do not respond to conventional therapy or are intolerant (Rubin et al., 2019; Raine et al., 2022). Biosimilars are biological agents with similar therapeutic effects to approved reference drugs regarding quality, efficacy, and safety. Biosimilars have great potential for cost savings and extraordinary accessibility (Kim et al., 2020). The first biosimilar for IFX (CT-P13) was available in 2013, whereas ADA biosimilars have been licensed since 2017. ADA biosimilar HS016 used in this study has completed a phase III clinical trial in patients with ankylosing spondylitis (AS) (Su et al., 2020). The results showed similar efficacy and safety compared with the ADA originator (Humira). Then it was validated by antibody-dependent cell-mediated cytotoxicity (ADCC) and complement-dependent cytotoxicity (CDC) activity (Gao et al., 2022).

Consequently, HS016 became China’s first ADA biosimilar approved for CD indications. Several randomized clinical trials, including CLASSIC-I, CHARM, ULTRA 1, and ULTRA 2 (Hanauer et al., 2006; Colombel et al., 2007; Reinisch et al., 2011; Sandborn et al., 2012), demonstrated the efficacy and safety of ADA in moderate to severe CD and UC treatment. Multiple real-world data have also confirmed the effectiveness of ADA in CD and UC therapy (Sohn et al., 2016; Moens et al., 2022; Vitello et al., 2022). ECCO and AGA guidelines also propose that the safety and efficacy of the original ADA and biosimilars are consistent. Still, the biosimilar of ADA in China (HS016) is different from the ADA originator or the biosimilars in Europe and the United States (SB5, BI95501) (Derikx et al., 2021; Hanauer et al., 2021).

Although anti-TNFα unveils a new treatment for IBD, up to 30% of patients show primary nonresponse (PNR), and another 40% lose response over time, i.e., secondary loss of response (SLR) and need to switch therapy (Papamichael et al., 2015). So far, these patients obtained no benefit, are caused by inadequate drug concentrations or an increased drug clearance (Papamichael and Cheifetz, 2017). Therapeutic drug monitoring (TDM) has already become a practical tool for the therapeutic management and optimization of anti-TNFα agents, including measuring serum and anti-drug antibody concentrations. Thus, serum drug concentrations have already been used as one of the biologics efficacy indicators. Higher serum drug concentrations are usually considered an objective outcome of treatment, while the lower suggest shortening dosing intervals or switching the treatment method. In comparison, anti-drug antibodies are a factor in lower drug concentration, so it is necessary to add immune suppressants or convert therapy (Papamichael et al., 2015; Vaughn et al., 2015).

In this study, we retrospectively analyzed the real-world data of HS016 in IBD treatment from one of the biggest IBD centers in China. In addition, we further discussed the factor (TDM) on the efficacy of HS016, providing more evidence and suggestions for clinical decision-making.

## 2 Materials and methods

### 2.1 Study subjects

This study was approved by the Medical Ethics Committee of the First Affiliated Hospital of Fourth Military Medical University, Xi’an, China (KY20222333-C-1). It was performed according to the ethical principles for medical research of the World Medical Association Declaration of Helsinki. All participants signed and informed consent.

One hundred three patients were considered for IBD treated with HS016 at Xijing Hospital (Xi’an, China) from October 2020 to April 2022. ADA is prescribed for patients (Kaplan, 2015) failed to respond to traditional treatment; (Torres et al., 2017) continued ulcer occurrence by endoscope; (Romagnani, 1999); after surgery for CD to prevent recurrence. It has been proved that the early use of biologics is beneficial to improving the recovery rate and reducing the operation and complications. In this study, HS016 was used for the first two types of participants.

**FIGURE 1:**
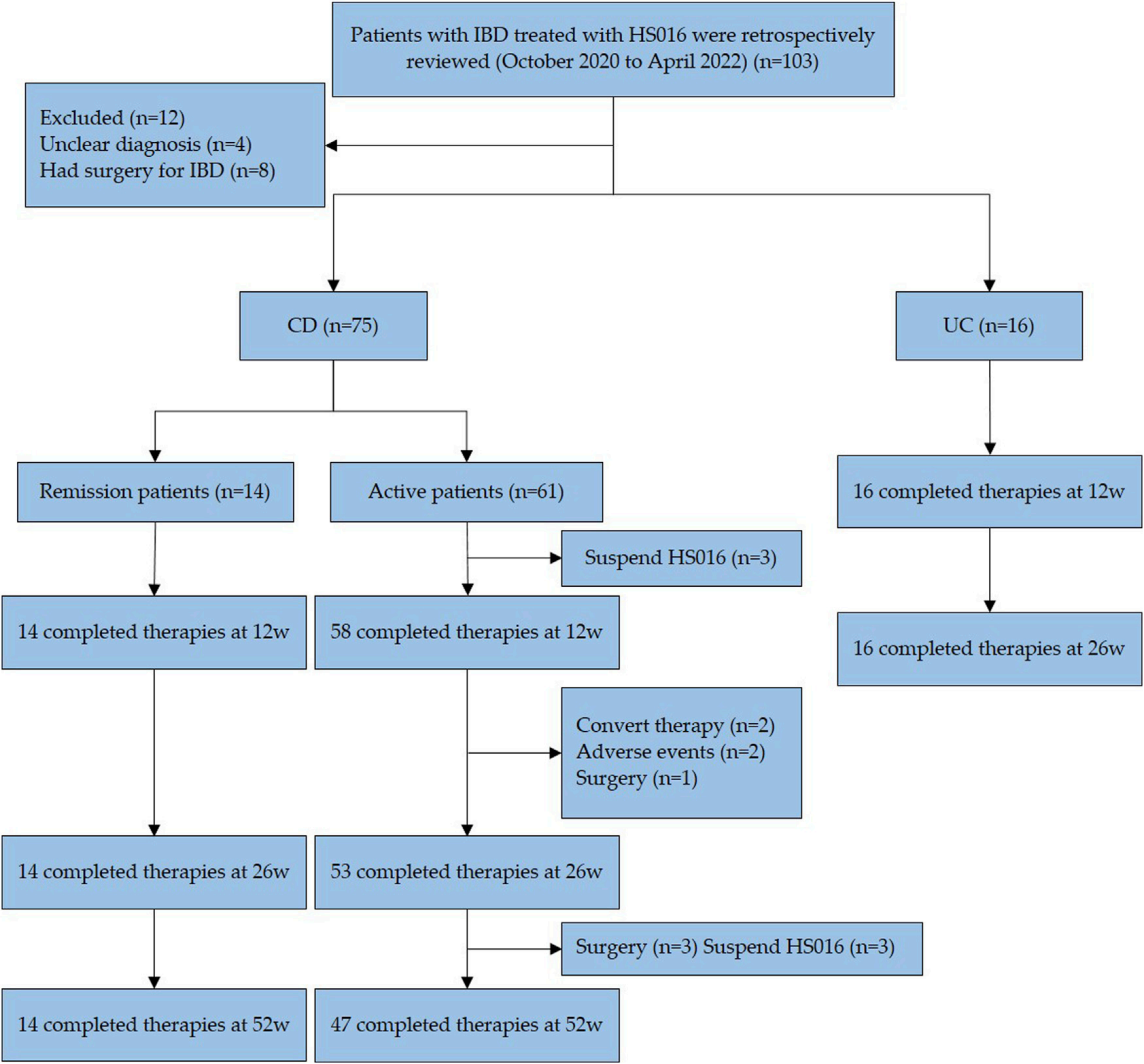
Flow diagram of patient selection. A total of 103 patients received treatment with HS016 during this period, 91 patients, met the inclusion and exclusion criteria, entered the study. 83 patients completed the 12-week and 26-week follow-ups, and 61 patients completed the 52-week follow-up. All data in this study were obtained from the patients mentioned above. IBD, inflammatory bowel disease; HS016, adalimumab biosimilar, CD, Crohn’s disease; UC, ulcerative colitis.

Patients should be excluded (Kaplan, 2015) if they had an ulcer (s) that cannot be distinguished from intestinal Behcet’s disease, intestinal tuberculosis, or unspecified types of colitis; (Torres et al., 2017) due to nonclinical factors (factors other than ineffectiveness, failure to respond, or intolerance) would be terminated; (Romagnani, 1999) IBDs who were preparing for surgery; (Pouillon et al., 2020) IBDs who were pregnant or preparing for pregnancy; (Tracey et al., 2008) Any condition was considered that prevented completion of the research or interferes with the result analysis, including patients with a history of drug or alcohol abuse, mental illness or poor compliance, definite immune system disorders or hematologic difficulties, or carcinoma. Among these 103 participants, 12 were excluded because they could not make a definitive diagnosis (*n* = 4) and had had IBD surgery (*n* = 8). The remaining 91 patients (75 CD and 14 UC) met the study criteria and were enrolled in this retrospective analysis (Figure 1). IBD diagnosis was based on endoscopy and pathological biopsy (Rubin et al., 2019; Raine et al., 2022).

### 2.2 Treatment and evaluation

In this study, demographic data (e.g., gender, age, height, weight) and clinical data (e.g., disease duration, previous medications, disease typing, extraintestinal manifestations, complications of IBD, previous surgical history, endoscopic and pathological findings) were collected from patients.

Patients with IBD were treated with ADA biosimilar HS016 (Anjianning; Hisun Bio-pharmaceutical Co., Ltd.), a 160 mg dose at the baseline, an 80 mg dose at week 2, then a 40 mg dose every other week. In case of insufficient efficacy during treatment, the dose would be increased to 40 mg per week after the physician’s judgment.

The HS016 efficacy assessment of CD patients by Harvey-Bradshaw Index (HBI) and Simplified Endoscopic Score for Crohn’s Disease (SES-CD) and UC by partial Mayo score (pMS). Patients were evaluated at weeks 0, 12, 26, and 52. An HBI score of ≤4 points was defined as remission, 5–7 points mildly active, 8–16 points moderately active, and >16 points severely active. Clinical response was defined as a decrease in HBI score ≥3 points after treatment; clinical remission was defined as an HBI score ≤4 points, and maintenance of clinical remission was defined as a sustained HBI score ≤4 points before and after treatment with ADA biosimilar (Harvey and Bradshaw, 1980; Daperno et al., 2004). In UC, pMS with 2–4 points being classified as mild, 5–7 points as moderate, and 7–9 points as severe; clinical remission was defined as pMS <2 points, clinical response was defined as the decrease of pMS ≥30% and ≥3 points compared to baseline values or with a reduction of ≥1 points in the score item for blood in stool (Lewis et al., 2008). Regarding endoscopic efficacy, the response was defined as a ≥50% decrease in SES-CD of CD or Mayo score ≤1 point of UC. And endoscopic remission was described as an SES-CD score ≤3 Points or no visible ulcer at endoscopy of CD and Mayo score equal to 0 points no visible ulcer at endoscopy of UC [17, 24]. Side effects in all patients who received at least one dose of HS016 were recorded, such as reactions to injection, acute or chronic infections, and tumors.

### 2.3 Determination of therapeutic drug monitoring

Serum TNF-α, drug concentration, and anti-ADA biosimilar antibody were measured by immunochromatography in patients with persistent non-benefit under HS016 therapy, either clinical or endoscopic. Suzhou HeRui Biotechnology Company carries out serum TNF-α and TDM. The reference range of serum TNF-α is less than 8.1 pg/mL, the reference range of therapeutic drug concentration is ≥5.0 μg/mL, and the reference range of anti-ADA biosimilar antibody is under 4 ng/mL (Suzhou HeRui Biotechnology Co., Ltd. Suzhou, China).

### 2.4 Statistical analysis

Data were presented as mean ± SD. Statistical analysis was performed by using the Students t-test (Mann Whitney for data not normally distributed) with IBM SPSS (v.26.0) and GraphPad Prism (v.8.3.0). Rates were compared by χ^2^ test or Fisher’s exact probability method. Comparing the means of two or more groups (independent variables) on a single dependent variable using one-way ANOVA. Pearson correlation analysis was performed to analyze the correlation between the serum TNF-α concentrations and disease severity. We used the SES-CD score to represent disease severity. All studies used 2-sided tests. The significant differences were documented as *p* < 0.05.

## 3 Results

### 3.1 Baseline characteristics of the study subjects

This study has 91 patients with IBD (75 CD, 16 UC) (Figure 1). A total of 77 IBD patients completed the 52-week follow-up. 3 CD patients discontinued treatment before 12-week treatment, voluntarily stopping their medication. 5 CD patients discontinued treatment before 26-week treatment, with reasons including convert therapy (*n* = 2), adverse events (*n* = 2), and surgery (*n* = 1). 6 CD patients discontinued treatment before 52-week treatment, with reasons including medication ineffectiveness (*n* = 3) and surgery (*n* = 3). According to the HBI score at baseline, CD patients were divided into a clinical remission group and an activity group to assess. Among these 75 patients, 14 were in clinical remission, which showed differences in disease sites from active patients at baseline (*p* = 0.013). Of all CD patients, twenty-two had extraintestinal manifestations, including seven arthritis, eleven oral ulcers, five perianal abscesses, one anal fistula, one iritis, and one erythema nodosum. Among eight patients exposed previously to TNF-antagonist, three discontinued due to secondary loss of response, one due to high antibody concentration, and four due to allergy. These patients had received treatment with Infliximab before using HS016. Infliximab is a human-murine chimeric antibody, while HS016 is a fully humanized antibody, patients who experienced allergy with Infliximab were advised to switch to the safer HS016 for treatment. Of the 16 UC patients with a median age of 40.5 years, 11 (68.8%) were male, and 11 manifested extensive colitis. Four patients (25%) were mildly active, three patients (18.8%) were moderately active, and nine patients (56.2%) were severely active according to the partial Mayo score (pMS) at baseline. Previously 75% of patients were on 5-ASA, 50% on steroids, and 31.2% on infliximab, as shown in Table 1.

**TABLE 1:** Baseline characteristics of all patients.

	CD (*n* = 75)		*p*	UC (*n* = 16)
	CD in clinical remission at baseline ^a^ (*n* = 14)	CD in active at baseline ^b^ (*n* = 61)		
Male [n (%)]	10 (71.4%)	41 (67.2%)	1.000	11 (68.8%)
Age [y, M(Q1, Q3)]	30.5 (21.75, 41)	33 (23, 46)	0.492	40.5 (31.5, 56.8)
Location of disease [n (%)] (CD)				
L1	7 (50.0%)	12 (19.7%)	0.013	
L2	1 (7.1%)	25 (41%)		
L3	5 (35.7%)	23 (37.7%)		
L4	1 (7.1%)	1 (1.6%)		
Disease behavior [n (%)] (CD)			0.632	
B1	10 (71.4%)	36 (59.0%)		
B2	4 (28.6%)	24 (39.3%)		
B3	0	1 (1.6%)		
Location of disease [n (%)] (UC)				
E1				2 (12.5%)
E2				3 (18.8%)
E3				11 (68.7%)
Previous medications [n (%)]				
5-Aminosalicylic acid	3 (21.4%)	29 (47.5%)	0.075	12 (75.0%)
Corticosteroids	2 (14.3%)	16 (26.2%)	0.496	8 (50.0%)
Immunosuppressant	2 (14.3%)	4 (6.6%)	0.311	5 (31.2%)
Biologics	2 (14.3%)	6 (9.8%)	0.638	5 (31.2%)
Anti-tuberculosis therapy	0	6 (9.8%)	0.586	
Previous surgery [n (%)]	1 (9.8%)	3 (4.9%)	0.571	
Baseline level [n (%)]			<0.001	
Clinical remission	14 (100%)	0		0
Mildly active	0	30 (49.2%)		4 (25.0%)
Moderately active	0	30 (49.2%)		3 (18.80%)
Severely active	0	1 (1.6%)		9 (56.20%)
HBI/PMS score [M(Q1, Q3)]	2 (2, 4)	8 (6, 9)	<0.001	8 (3, 8)

Comparing the baseline data of patients in clinical remission and clinical active, except for differences in baseline disease activity (*p* < 0.001), there were no statistically significant differences in other indicators (*p* > 0.05). Data were presented as mean ± SD. Statistical analysis was performed by using the Students t-test (Mann Whitney for data not normally distributed). Rates were compared by χ2 test or Fisher′’s exact probability method. CD, Crohn’s disease; UC, ulcerative colitis; L1, terminal ileum; L2, colon; L3, ileum colon; L4, upper gastrointestinal tract; B1, Non-narrow, non-fistula; B2, narrow; B3, fistula; E1, proctitis; E2, Left-sided colitis; E3, Extensive colitis.

^a^ CD in clinical remission at baseline means HBI score ≤4 points but still have ulcers observed during endoscopy at baseline.

^b^ CD in clinical active at baseline means HBI score >4 points at baseline.

### 3.2 Efficacy

**FIGURE 2:**
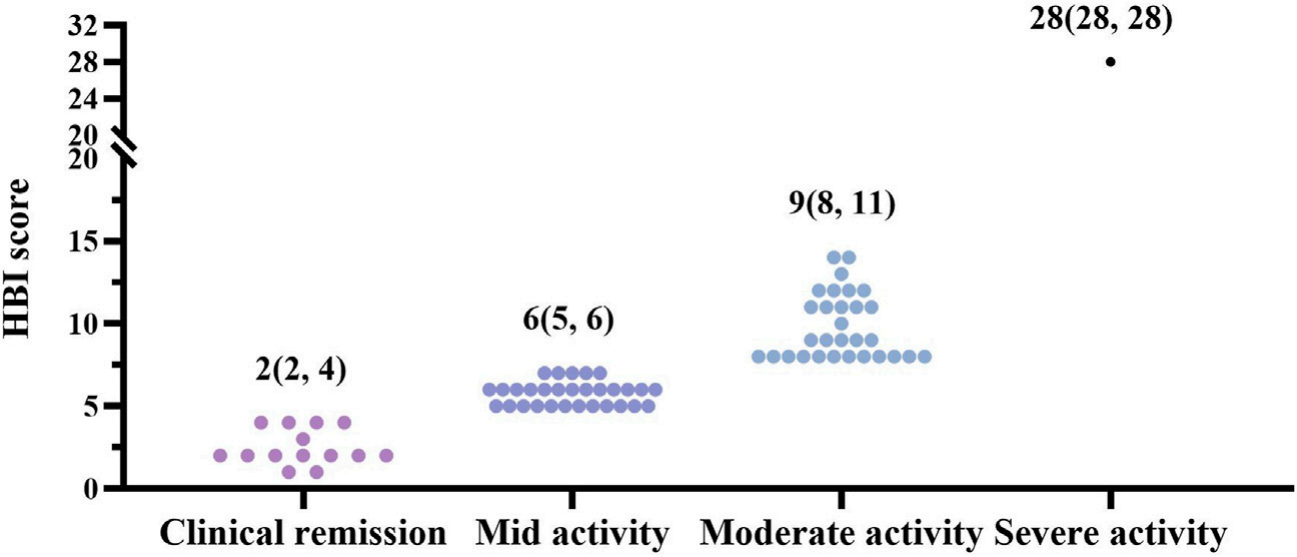
Distribution of disease severity in CD patients based on HBI scores at baseline. Crohn’s disease patients were divided into different groups based on disease severity, as HBI score, 0–4 points as clinical remission (*n* = 14, 18.7%), 5–7 points as mid active (*n* = 30, 40%), 8–16 points as moderate activity (*n* = 30, 40%), and >16 points as severe activity (*n* = 1, 1.3%) (Reinisch et al., 2011). 98% of active patients were mild to moderate (*n* = 60). CD, Crohn’s disease; HBI, Harvey-Bradshaw Index.

**FIGURE 3:**
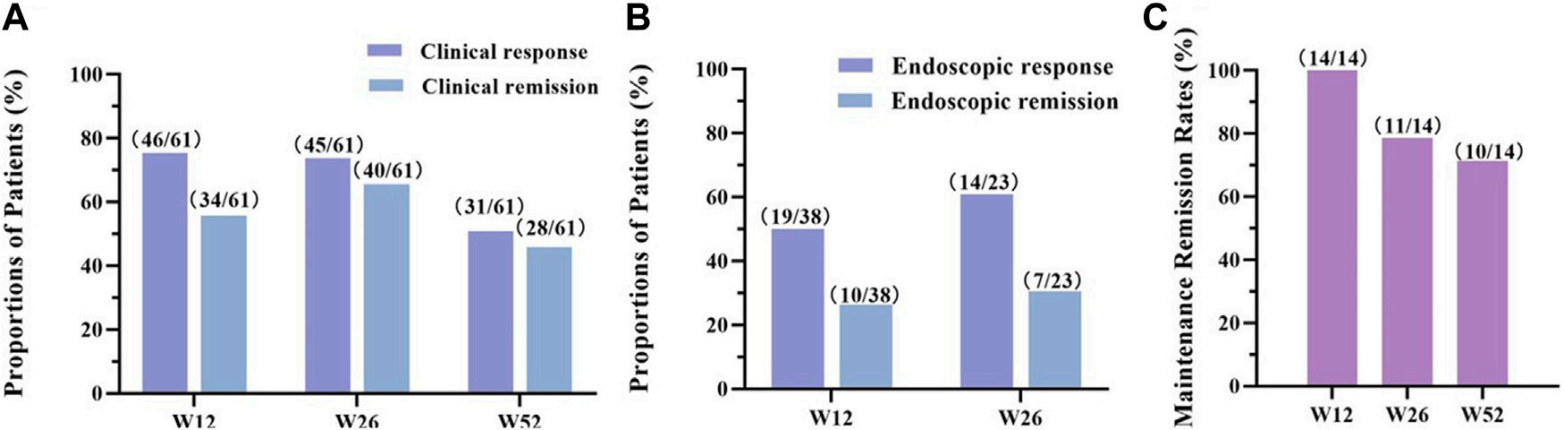
The treatment outcomes of CD patients. **(A)** The clinical response and clinical remission rates among active CD patients. After 12-week, 26-week, and 52-week treatment, patients’ HBI scores were assessed and documented as clinical response (HBI score decrease ≥3 points) and clinical remission (HBI score ≤4 points); **(B)** The endoscopic response and endoscopic remission rates among active CD patients. After 12 w and 26 w of treatment, patients’ SES-CD scores were assessed and documented as endoscopic response (SES-CD score decrease ≥50%) and endoscopic remission (SES-CD score ≤3 points or “no visible ulcer” at endoscopy); **(C)** The clinical maintenance remission rates of CD patients. After 12-week, 26-week, and 52-week treatment, patients’ HBI scores were assessed. The patients, whose HBI score ≤4 points during every assessment, were documented as clinical maintenance remission. CD, Crohn’s disease; HBI, Harvey-Bradshaw Index; SES-CD, Simplified Endoscopic Score for Crohn’s Disease.

We first assessed the distribution of baseline levels in all CD patients according to Harvey-Bradshaw Index (HBI) scores. 81.3% of CD patients were in the active phase, and most were mild to moderately active (Figure 2). During our follow-up visits, three active CD patients were discontinued before completing 12 weeks of treatment for personal reasons; the other five patients uncompleted 26-week therapy as a lost response (Figure 1). In our study, the clinical response rates with active CD were 75.4% (46/61) at 12 w, 73.8% (45/61) at 26 w, and 50.8% (31/61) at 52 w, respectively. 55.7% (34/61) at 12 w, 65.6% (40/61) at 26 w and 45.9% (28/61) at 52 w active CD patients achieved clinical remission (Figure 3A). Dou to the COVID-19 pandemic during the follow-up period, the patients were unable to follow-up or unwilling to undergo invasive examinations. So we obtained 38 patients’ endoscopy at 12w and 23 patients’ endoscopy at 26 w (Tables 2, 3). The endoscopic response rates were 50% (19/38) at 12 w. 26.3% (10/38) attained endoscopic remission, and all of them also reached mucosal healing. The endoscopic response and remission were 60.9% (14/23) and 30.4% (7/23) at 26 weeks. Particularly, 21.7% (5/23) got mucosal healing (Figure 3B). Among the 14 patients who entered the maintenance remission phase, before HS016 treatment, 3 patients used 5-ASA, 2 patients tapered off steroids, 2 patients used immunosuppressants, and 2 patients were treated with infliximab The maintained remission rates of 14 CD patients in remission were 100% (14/14) at 12 w, 78.6% (11/14) at 26 w, and 71.4% (10/14) at 52 w (Figure 3C). At 12 w, 3 patients showed endoscopic response, and 1 patient achieved endoscopic remission. At 26 w, 7 patients showed endoscopic response, and 3 patients achieved endoscopic remission. The efficacy of patients who had received Infliximab before using HS016 were analyzed. Among these patients who experienced secondary loss of response to Infliximab, after 52-week treatment, one patient achieved clinical remission, one patient showed no response to treatment, and another patient initially responded well but developed secondary loss of response. Among the four patients who experienced allergy to Infliximab, one patient maintained remission, two patients achieved clinical remission, and one patient showed no response to treatment. Another patient who developed antibodies while receiving treatment with Infliximab showed initial effectiveness. However, after 52-week treatment, he experienced clinical nonresponse to HS016.

**TABLE 2:** Baseline of CD patients undergoing endoscopy after 12-week treatment.

		CD (*n* = 38)		
	All	CD in endoscopic non-response ^a^ (*n* = 19)		CD in endoscopic response ^b^ (*n* = 19)
Male [n (%)]	35 (65.8%)	12 (63.2%)		13 (68.4%)
Age [y, M(Q1, Q3)]	34 (22.5, 46.3)	36 (24, 46)		33 (20, 48)
Location of disease [n (%)]				
L1	4 (10.5%)	1 (5.3%)		3 (15.8%)
L2	15 (39.5%)	10 (52.6%)		5 (26.3%)
L3	19 (50%)	8 (42.1%)		11 (57.9%)
L4	0	0		
Disease behavior [n (%)]				
B1	23 (60.5%)	8 (42.1%)		15 (78.9%)
B2	15 (39.5%)	11 (57.9%)		4 (21,1%)
B3	0	0		0
Previous medications [n (%)]				
5-Aminosalicylic acid	20 (52.6%)	9 (47.4%)		11 (57.9%)
Corticosteroids	10 (26.3%)	6 (31.6%)		4 (21.0%)
Immunosuppressant	3 (7.9%)	2 (10.5%)		1 (5.3%)
Biologics	3 (7.9%)	3 (15.8%)		0
Anti-tuberculosis therapy	2 (5.3%)	1 (5.3%)		1 (5.3%)
HBI/PMS score [M(Q1, Q3)]	7 (5, 9.3)	8 (6, 8)		7 (5, 11)
SES-CD score [M(Q1, Q3)]	11.5 (7,16)	11 (7,13)		13 6, 20)

CD, Crohn’s disease; UC, ulcerative colitis; L1, terminal ileum; L2, colon; L3, ileum colon; L4, upper gastrointestinal tract; B1, Non-narrow, non-fistula; B2, narrow; B3, fistula; E1, proctitis; E2, Left-sided colitis; E3, extensive colitis; HBI, Harvey-Bradshaw Index; SES-CD, Simplified Endoscopic Score for Crohn’s Disease.

^a^ CD in endoscopic non-response means after 12-week treatment, patients’ SES-CD scores were assessed and documented as endoscopic non-response (SES-CD, score decrease <50%).

^b^ CD in endoscopic response means after 12-week treatment, patients’ SES-CD scores were assessed and documented as endoscopic response (SES-CD score decrease ≥50%).

**TABLE 3:** Baseline of CD patients undergoing endoscopy after 26-week treatment.

	CD (*n* = 23)			
	All	CD in endoscopic non-response ^a^ (*n* = 9)	CD in endoscopic response ^b^ (*n* = 14)	
Male [n (%)]	16 (69.6%)	7 (77.8%)	9 (64.3%)	
Age [y, M(Q1, Q3)]	34 (28, 44)	40 (29, 47)	33.5 (23.8, 40.8)	
Location of disease [n (%)]				
L1	4 (17.4%)	1 (4.3%)	3 (21.4%)	
L2	11 (47.8%)	7 (77.8%)	4 (28.6%)	
L3	8 (34.8%)	1 (4.3%)	7 (50%)	
L4	0	0		
Disease behavior [n (%)]				
B1	12 (52.2%)	4 (44.4%)	8 (57.1%)	
B2	10 (43.5%)	4 (44.4%)	6 (42.9%)	
B3	1 (4.3%)	1 (4.3%)	0	
Previous medications [n (%)]				
5-Aminosalicylic acid	11 (47.8%)	3 (33.3%)	8 (57.1%)	
Corticosteroids	9 (39.1%)	3 (33.3%)	6 (42.9%)	
Immunosuppressant	4 (17.4%)	3 (33.3%)	1 (5.3%)	
Biologics	2 (8.7%)	1 (15.8%)	0	
Anti-tuberculosis therapy	2 (8.7%)	1 (4.3%)	1 (7.1%)	
HBI/PMS score [M(Q1, Q3)]	6 (5, 8)	6 (5.5, 8.5)	6 (4.3, 8.3)	
SES-CD score [M(Q1, Q3)]	8 (7, 13)	8 (6.5, 11)	11 (7.5, 13.3)	

CD, Crohn’s disease; UC, ulcerative colitis; L1, terminal ileum; L2, colon; L3, ileum colon; L4, upper gastrointestinal tract; B1, Non-narrow, non-fistula; B2, narrow; B3, fistula; E1, proctitis; E2, Left-sided colitis; E3, extensive colitis; HBI, Harvey-Bradshaw Index; SES-CD, Simplified Endoscopic Score for Crohn’s Disease.

^a^ CD in endoscopic non-response means after 26-week treatment, patients’ SES-CD scores were assessed and documented as endoscopic non-response (SES-CD, score decrease <50%).

^b^ CD in endoscopic response means after 26-week treatment, patients’ SES-CD scores were assessed and documented as endoscopic response (SES-CD score decrease ≥50%).

**FIGURE 4:**
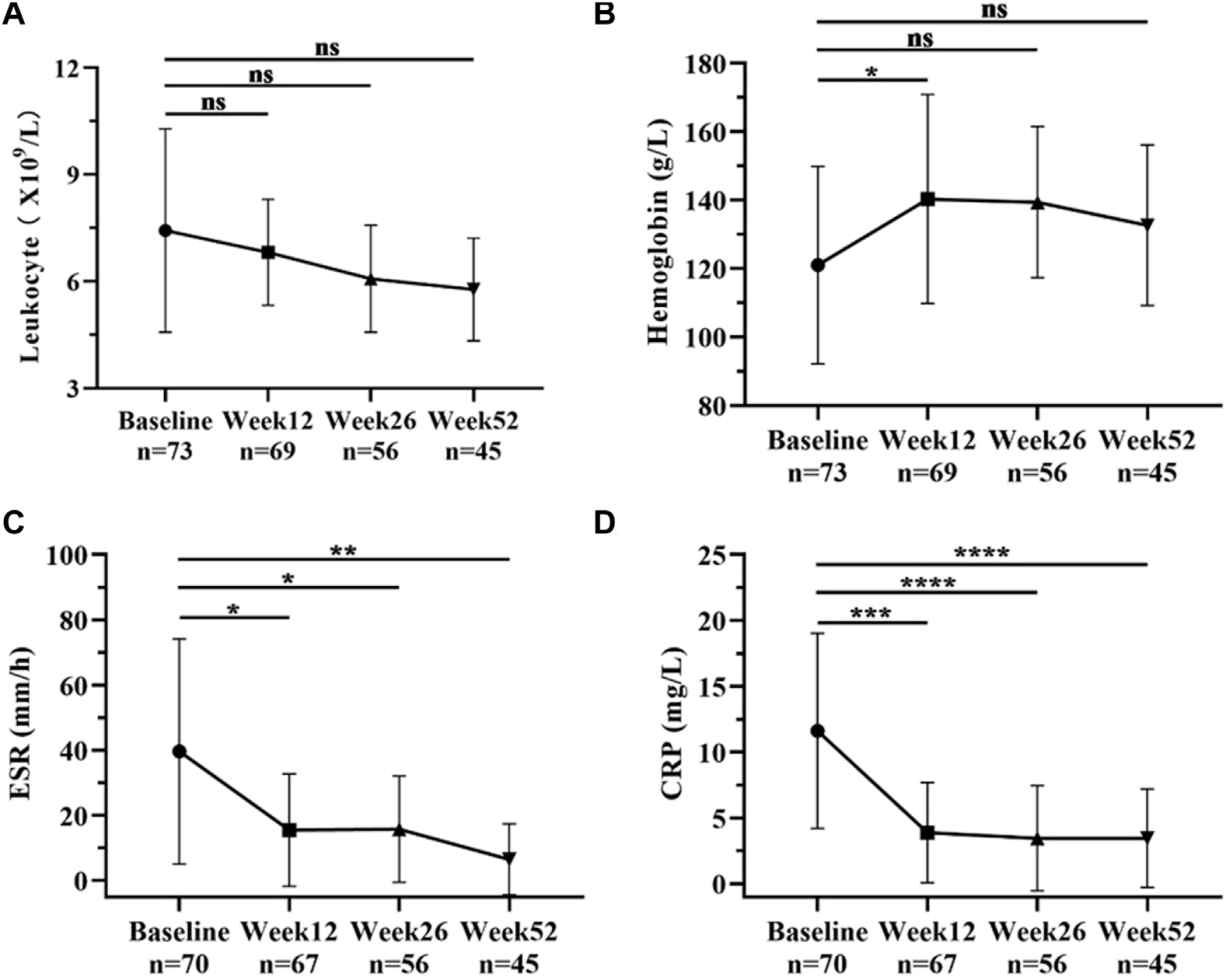
CD patients’ change of every index was observed at baseline, week 12, week 26, and week 52. **(A)** Change of leukocytes among CD patients at baseline, week 12, week 26, and week 52; **(B)** Change of hemoglobin among CD patients at baseline, week 12, week 26, and week 52; **(C)** Change of ESR among CD patients at baseline, week 12, week 26, and week 52; **(D)** Change of CRP among CD patients at baseline, week 12, week 26, and week 52. The data represent the mean ± SD *p < 0.05, **p < 0.01, ****p < 0.001, ns, *p* > 0.05 by one-way ANOVA. CRP, C-reactive protein; ESR, erythrocyte sedimentation rate.

Additionally, CD patients’ change of every index was analyzed at baseline, week 12, week 26, and week 52. (Figure 4) However, the incidence of primary nonresponse was 12.0% (9/75), and secondary loss of response was 22.7% (17/75) within 52 weeks of ADA biosimilar follow-up with CD. Of these, seven patients underwent surgery due to unrelieved after treatment.

As for the UC patients, 37.5% (6/16) gained a clinical response at a 12-week follow-up. The clinical response and remission at 26 w were 56.3% (9/16) and 31.3% (5/16). At 12 w, the endoscopic response was 50% (4/8), and remission was 37.5% (3/8). The endoscopic remission rate was 42.8% (3/7) with 26-week treatment. During the follow-up, two patients had surgeries.

### 3.3 Results of TDM

**FIGURE 5:**
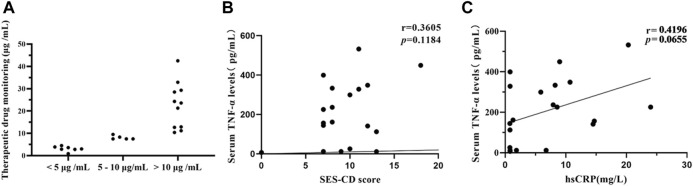
TDM and serum TNF-α concentration were analyzed among portal CD patients. Patients who did not respond after a minimum of 12-week treatment underwent TDM and serum TNF-α concentration **(A)** Therapeutic drug concentration in 23 patients. 23 patients included 9 patients with primary non-response, 9 patients who did not show response under endoscopy at the 26-week follow-up, and 5 patients who did not achieve clinical remission at the 26-week follow-up. **(B)** Correlation analysis between serum TNF-α concentration and SES-CD score in 20 patients. The results indicate that there is no correlation between serum TNF-α concentration and SES-CD score. (r = 0.3605, *p* = 0.1184) **(C)** Correlation analysis between serum TNF-α concentration and CRP in 20 patients. (r = 0.4196, *p* = 0.0655). CD, Crohn’s disease; TDM, Therapeutic drug monitoring; SES-CD, Simplified Endoscopic Score for Crohn’s Disease; CRP, C-reactive protein.

Considering the feasibility of TDM, only a portion of patients were willing to undergo the drug concentration and anti-ADA biosimilar antibodies examination (*n* = 23), including the 9 patients with primary non-response, 9 patients who did not show response under endoscopy at the 26-week follow-up, and 5 patients who did not achieve clinical remission at the 26-week follow-up. 7 (30.4%) patients had drug concentrations less than 5 μg/mL, 5 (21.74%) patients between 5 and 10 μg/mL, and 11 (47.8%) patients above 10 μg/mL (Figure 5A). One additional patient had tested anti-drug antibodies in the serum. Only a tiny percentage of patients (34.8%) who failed to respond had lower therapeutic drug concentration or anti-drug antibodies developing.

### 3.4 Correlation between serum TNF-α and disease severity

20 patients, who were not endoscopy response after a minimum of 12-week treatment, underwent serum TNF-α quantification, subject to the patients’ consent. The endoscope and CRP during the same period (no more than 1 week before and after) were also recorded of these patients. And then, we performed a Pearson correlation analysis of two targets. The results showed that there was only a weak correlation between serum TNF-α and SES-CD (r = 0.3605, *p* = 0.1184). (Figure 5B). Additionally the results showed that there was only a weak correlation between serum TNF-α and CRP (r = 0.4196, *p* = 0.0655) (Figure 5C).

### 3.5 Safety

There were 3 cases of psoriasis-like rash. Of these, one developed a large rash on the forehead and back after the initial treatment, and the other 2 underwent scattered rashes on the extremities at 12 and 52 weeks. In addition, there were two cases of fungal otitis externa. Other serious adverse events were not identified in our study.

## 4 Discussion

The primary outcomes of this study show that HS016 has a significant therapeutic effect on IBD patients, and the most frequent factor for the ineffective HS016 treatment is insufficient serum concentration. Our research proves to physicians that HS016 is a practical and safe treatment option for IBD.

Compared to IFX, ADA is a fully human-originated antibody with lower immunogenicity and higher safety. The subcutaneous injection mode of administration makes its application more and more widespread. A large number of studies have demonstrated the efficacy of ADA for IBD. For example, CLASSIC- I (Hanauer et al., 2006) reported a clinical remission rate of 36% at 4 weeks with ADA in CD patients, which was significantly better than the placebo-controlled group. CLASSIC- II (Sandborn et al., 2007) showed induction remission of CD was 46% at 56 weeks. In addition, several domestic and international studies (Derikx et al., 2021; Hanauer et al., 2021; Tursi et al., 2022) affirmed the efficacy of other ADA biosimilars (SB5, BI95501), including IBD patients initially treated with ADA biosimilar and in conversion from the original ADA. EXTEND (Rutgeerts et al., 2012) indicated that the mucosal healing rates among ADA-treated CD patients were 27% at 12 w and 24% at 52 w, respectively. However, there are no relevant data on the efficacy of HS016 in China, and our study confirmed its effectiveness and safety. Our results were similar to or better than the present studies.

On the one hand, the efficacy of HS016 is confirmed. On the other hand, 98% of CD patients in our study were mild to moderate. It also shows that early application of biologics can improve the cure rates of IBD patients. All findings above suggested that ADA biosimilar HS016 showed similar efficacy as the original ADA.

Moens et al. (Moens et al., 2022) also revealed that ADA for UC had a clinical remission rate of 26% at 26 weeks. At the same time, the results of Iborra M et al. (Iborra et al., 2017) showed clinical remission rates of 26% and 43% at 12 and 26 weeks, respectively. Although ADA is not often used to treat UC in China (due to China’s FDA policies), ECCO, ACG (Rubin et al., 2019; Raine et al., 2022) have clarified that ADA can be used for the treatment of moderately severe UC in adults, especially those with inadequate, intolerant, or contraindicated response to glucocorticoid therapies. Compared to the studies above, our study’s lower clinical remission rates (31.3% at 26 w) may be mainly due to the sample size and a high proportion of patients with severe inflammation (56.9%) at baseline. Therefore, further investigation with more data is still needed.

Regarding safety, PYRAMID (DʼHaens et al., 2018) with up to 6 years of follow-up, confirmed the safety of ADA, which did not increase the risk of various side effects, such as lymphoma. A meta-analysis (Singh et al., 2011) included 160 randomized controlled studies and pointed out no additional risk of overall or severe adverse events, serious infections, or withdrawal study due to adverse events compared to the placebo. The incidence of ADA biosimilar adverse events, reported in the VOLTAIRE-CD (Hanauer et al., 2021), showed that the most common occurring infection (24%) and injection reaction (23%) rates were similar to ADA originator. The occurrence of side effects in IBD patients treated with HS016 was 5.5% (5/91), including psoriatic-like rash and fungal otitis externa, which had also been reported with the original ADA. Moreover, Cao et al. discovered that an allergic reaction, which appeared in a patient because of the original drug, would recover after conversion to ADA biosimilar (Cao et al., 2022). Although it is a case report, it may still demonstrate biosimilars is safer than original drug in certain aspects.

Anti-TNFα biologics mainly treat IBD by inactivating inflammatory factors, thus inhibiting inflammation (Tracey et al., 2008; Allez, 2012). Our study analyzed the correlation between serum TNF-α levels and disease severity (SES-CD/CRP) in 20 CD patients treated with HS016 for ≥ 3 months. The results showed no significant correlation between serum TNF-α levels and the severity of the disease. However, all 20 patients’ serum TNF-α levels remained elevated after therapy; some were in clinical remission. Additionally, a previous study (Martínez-Borra et al., 2002) concluded that the serum levels of TNF-α are associated with the response to infliximab and could help to identify patients who would benefit from anti–TNFα treatment. But more studies (Louis et al., 2002; Amini Kadijani et al., 2017; Billiet et al., 2017) showed the opposite. In our study, most patients tested for serum TNF-α concentrations, were subject to persistent endoscopic nonresponse after HS016 application, which may impact the analysis of the results. And only one time point of serum TNF levels is available for this analysis. Thus, the significance of serum TNF-α in IBD diagnosis and treatment remains unclear, and further research is needed to investigate the changes in TNF levels before and after TNF treatment.

Although a wide range of studies have demonstrated that TNF-α inhibitors are effective therapies for IBD, there are still approximately 40% of patients will experience primary nonresponse (PNR) or secondary loss of response (SLR) (Argollo et al., 2020). In our data, PNR is 12.0%, and SLR is 22.7%, which is highly consistent with the previous findings. Then dose increasing or treatment switching can both be executed based on TDM. We further analyzed the reasons for failing to respond. On the one hand, only one (4.3%) patient showed no response, possibly due to the production of anti-antibodies, which is lower than reported data in others (Kennedy et al., 2019).

On the other hand, 7 (30.4%) patients may have had inadequate drug concentrations (the drug concentration < 5 μg/mL), resulting in loss of response. However, different studies have inconsistent recommendations for the recommended concentration of ADA. One study suggests that ADA concentration >4.9 μg/mL is beneficial for mucosal healing in CD patients (Roblin et al., 2014). Another study suggests that ADA concentration should be >7.1 μg/mL for mucosal healing (Ungar et al., 2016). In conclusion, regardless of the classification, TDM can only indicate that the inadequate drug concentration may be a possible reason for the poor treatment response in some patients. There are still some limitations when applying TDM within clinical practice, such as when to use TDM and how to interpret and apply the results correctly. Currently, reactive TDM is regarded as the standard of care, while proactive TDM is developed as a new therapeutic strategy to optimize anti-TNFα therapy in IBD. More data are still needed to define optimal drug concentration by well-designed prospective studies and RCT focusing on proactive TDM, especially during induction therapy (Papamichael et al., 2019; Papamichael et al., 2022).

## 5 Conclusion

In conclusion, we have confirmed the efficacy and safety of ADA biosimilar HS016 among Chinese IBD patients. In particular, the real-world data is equal to the previously reported RCT studies on the effectiveness of HS016 with Chinese CD patients. As research continues, we are confident that increasing biological agents will appear for IBD therapy. Additionally, biosimilars will stimulate competition in the market and have incredible potential to expand patient exposure to biologics in the context of treatment recommendations. Appropriate use of TDM will provide physicians with a more significant basis for selecting and optimizing treatment.

Herein, we demonstrate that HS016 may be an effective and safe option for Chinese IBD patients.

## Data Availability

The original contributions presented in the study are included in the article/Supplementary material, further inquiries can be directed to the corresponding authors.
